# Recurrent Intermittent Small Bowel Obstruction as an Initial Manifestation of Primary Intestinal Diffuse Large B‐Cell Lymphoma: A Case Report and Systematic Literature Review

**DOI:** 10.1155/cris/1085579

**Published:** 2026-10-14

**Authors:** Karl Nilsson Grip, Ann-Catrin Ekelund, Marcus Gustavsson, Olof Söderman, Jenny Nyqvist-Streng

**Affiliations:** ^1^ Department of Surgery Skaraborg Hospital Skövde Region Västra Götaland Sweden; ^2^ Medical Library Skaraborg Hospital Skövde Region Västra Götaland Sweden; ^3^ Department of Clinical Pathology Skaraborg Hospital Skövde Region Västra Götaland Sweden; ^4^ Department of Clinical Radiology Skaraborg Hospital Skövde Region Västra Götaland Sweden; ^5^ Department of Surgery Institute of Clinical Sciences Sahlgrenska Academy University of Gothenburg Gothenburg Sweden gu.se

**Keywords:** case report, lymphoma, PRISMA, small bowel obstruction

## Abstract

Primary intestinal diffuse large B‐cell lymphoma (DLBCL) is a rare gastrointestinal malignancy and an uncommon cause of small bowel obstruction (SBO). We report a 70‐year‐old man with no history of abdominal surgery who presented with recurrent episodes of abdominal pain, vomiting, and transiently resolving SBO. Serial computed tomography (CT) demonstrated a persistent transition point associated with an increasingly evident mesenteric soft tissue mass, initially suspected to represent a neuroendocrine tumor. After repeated presentations and temporary improvement with conservative management, exploratory laparotomy revealed a distal ileal tumor with central mesenteric involvement encasing major mesenteric vessels. Segmental small bowel resection with primary anastomosis was performed, although macroscopic mesenteric disease remained. Histopathological examination confirmed CD10‐positive DLBCL, and systemic treatment with six cycles of R‐CHOP was initiated. A structured search of MEDLINE and Embase identified 78 publications describing 87 adult cases of intestinal lymphoma presenting with SBO. DLBCL was the most frequently reported subtype, and the ileum was the most commonly specified anatomical location. However, recurrent obstruction with transient clinical and radiological improvement was rarely emphasized. This case demonstrates that temporary resolution of obstructive symptoms does not exclude a persistent pathological lead point. Recurrent SBO in a patient without previous abdominal surgery, particularly when serial imaging demonstrates evolving mesenteric or intestinal abnormalities, should prompt renewed investigation for an underlying malignancy, including intestinal lymphoma. Repeat cross‐sectional imaging, multidisciplinary reassessment, and individualized consideration of operative exploration may facilitate timely diagnosis while avoiding conclusions that transient improvement represents definitive resolution.

## 1. Introduction

Small bowel obstruction (SBO) is a common surgical emergency and accounts for ~20% of acute abdominal surgical presentations in high‐income countries [1]. It remains an important cause of morbidity and mortality, particularly when diagnosis or definitive treatment is delayed [1, 2].

In adults, postoperative adhesions are the leading cause, followed by hernias, whereas malignancy is a less frequent but well‐recognized etiology. Reviews estimate that malignancy accounts for roughly 5%–20% of SBO overall, while one narrative review reports that small‐bowel malignancies account for about 5%–10% of SBO cases [3].

Primary tumors of the small intestine are uncommon. Although the small bowel constitutes most of the length and mucosal surface of the gastrointestinal tract, cancers arising in the small intestine account for less than 5% of gastrointestinal cancers. Among small‐bowel malignancies, adenocarcinomas and neuroendocrine tumors are the predominant histologies, whereas lymphomas represent a minority but still clinically important subgroup [4].

Primary gastrointestinal lymphoma is itself rare, accounting for about 1%–4% of gastrointestinal malignancies. Within the small intestine, lymphoma has been reported to comprise ~15%–20% of small‐intestinal neoplasms, with the ileum being the most commonly involved segment [5]. A recent case report similarly described primary ileal DLBCL presenting with mechanical bowel obstruction [6]. Histopathologically, the vast majority of primary gastrointestinal lymphomas are of B‐cell lineage, and diffuse large B‐cell lymphoma (DLBCL) is the most common pathological subtype [5].

Lymphoma is an uncommon direct cause of bowel obstruction at presentation [7]. Imaging reviews note that lymphoma less commonly causes bowel obstruction than adenocarcinoma or carcinoid tumors, although aggressive subtypes—particularly DLBCL—may present with a bulky circumferential mass causing SBO. Reported obstruction rates in intestinal lymphoma series are ~5%–16%, indicating that SBO is a recognized but not dominant presentation [8]. These data indicate that SBO due to a small‐bowel tumor is uncommon, and SBO caused specifically by small‐intestinal B‐cell lymphoma is rarer still. Nevertheless, because B‐cell lymphomas—especially DLBCL—constitute the dominant histologic category among gastrointestinal lymphomas, they remain an important diagnostic consideration when a small‐bowel mass presents with obstruction [2].

Taken together, while malignant SBO is well recognized, recurrent transiently resolving obstruction caused by primary intestinal lymphoma remains poorly characterized and may lead to delayed diagnosis due to temporary response to conservative treatment [9, 10]. To our knowledge, no prior structured synthesis has specifically focused on intestinal lymphoma presenting as recurrent or acute SBO.

## 2. Case Presentation

A 70‐year‐old male farmer with no history of smoking, alcohol use, or hereditary disease presented to the emergency department with acute onset of lower abdominal pain, vomiting, and watery diarrhea. His medical history was notable for benign prostatic hyperplasia, hypertension, and previous prostate surgery. On examination, he was afebrile with normal vital signs. Abdominal examination revealed mild tenderness in the left lower quadrant and a reducible umbilical hernia. Laboratory investigations, including white blood cell count, were within normal limits. The patient reported no melaena, haematochezia, or other signs of gastrointestinal bleeding. A fecal occult blood test was not performed during the acute clinical course.

Initial computed tomography (CT) of the abdomen demonstrated dilated small bowel loops up to 4 cm with air–fluid levels and a transition point in the right lower quadrant, consistent with SBO (Figure 1). A right‐sided inguinal hernia containing fluid, but no clear bowel content, was also noted. A contrast study showed the passage of contrast into the colon, and the patient improved with conservative management, including nasogastric decompression and intravenous fluids. He was discharged after resolving symptoms.

**Figure 1:**
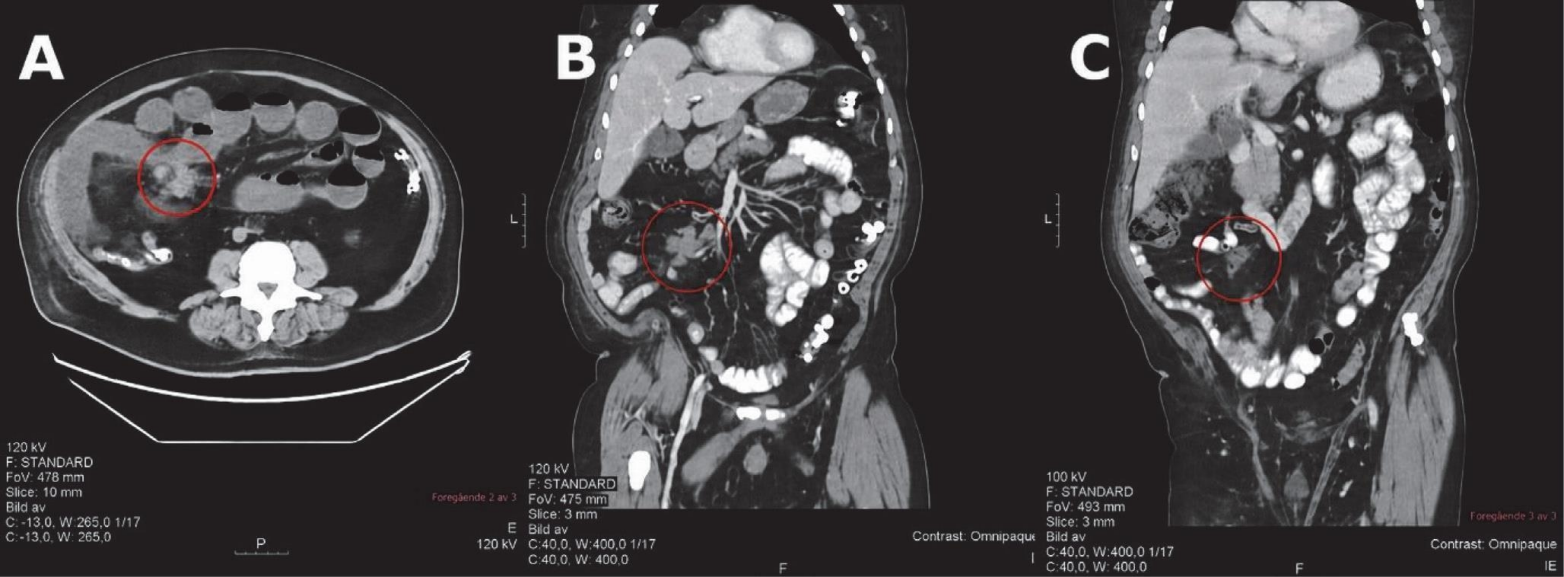
Radiological assessment of the abdominal tumor. (A) Axial reconstruction from a preoperative CT of the abdomen without intravenous contrast, demonstrating the tumor (circled) and its effect on the small bowel, which appears dilated with multiple air–fluid levels, consistent with ileus. (B) Coronal reconstruction from a preoperative contrast–enhanced CT of the abdomen performed for staging, demonstrating spontaneous regression of the ileus. The tumor remains visible (circled). (C) Coronal reconstruction from an acute contrast–enhanced CT performed due to abdominal pain, showing a marked partial regression in tumor size (circled).

Within days, the patient re‐presented with recurrent abdominal pain and vomiting. Repeat CT imaging again demonstrated SBO with a suspected transition point in the right abdomen. Notably, a 3 × 5 cm^2^ soft tissue mass was now identified in the right mesentery, raising the suspicion of a neuroendocrine tumor or lymph node metastasis. A subsequent small bowel contrast study showed persistent dilated loops without progression of contrast into the colon. Conservative management was again attempted, and the patient was discharged with plans for outpatient follow‐up.

Shortly thereafter, the patient returned with a third episode of abdominal pain and nausea, now accompanied by acute kidney injury, likely secondary to dehydration. Repeat CT imaging confirmed persistent SBO with a transition point associated with a mesenteric soft tissue mass in the right upper abdomen, again raising suspicion for malignancy.

Given the recurrent nature of the obstruction and imaging findings suggestive of a pathological lead point, an exploratory laparotomy was performed. Intraoperatively, a tumor was identified in the distal ileum with associated mesenteric involvement. A firm central mesenteric mass encasing the superior mesenteric artery (SMA) and ileocolic vessels was palpated. No peritoneal carcinomatosis was observed. A segmental small bowel resection of ~30 cm was performed with primary anastomosis. Because the central mesenteric component encased the superior mesenteric and ileocolic vessels, complete resection was not considered safe. Macroscopic residual disease remained in the central mesentery, corresponding to an R2 resection.

Upper gastrointestinal endoscopy and colonoscopy were not performed during the acute obstructive course because imaging localized the suspected lesion to the distal ileum and central mesentery, beyond the expected reach of conventional upper gastrointestinal endoscopy and not reliably accessible by colonoscopy. Because of the recurrent mechanical obstruction, operative exploration was prioritized to relieve the obstruction and obtain a histopathological diagnosis.

The postoperative course was uneventful, and the patient recovered well. Histopathological examination demonstrated DLBCL with a germinal‐centre B‐cell immunophenotype. The neoplastic cells were positive for CD20, CD10, and BCL6 and negative for BCL2, CD5, MUM1, c‐MYC, cyclin D1, TdT, and EBER. The Ki‐67 proliferation index was ~50%. Fluorescence in situ hybridization (FISH) demonstrated the loss of one copy of c‐MYC at 8q24 but no c‐MYC rearrangement. Macroscopic and histopathological findings supported a primary intestinal origin, with a dominant distal ileal tumor causing severe mural thickening and luminal stenosis and contiguous extension into the adjacent mesenteric tissue. Residual tumor was present in the central mesentery (Figure 2). Staging investigations, including CT of the thorax and bone marrow biopsy, showed no evidence of distant disease. No PET‐CT was performed. A formal Lugano stage was not documented and could not be assigned reliably in retrospect from the available clinical records.

**Figure 2:**
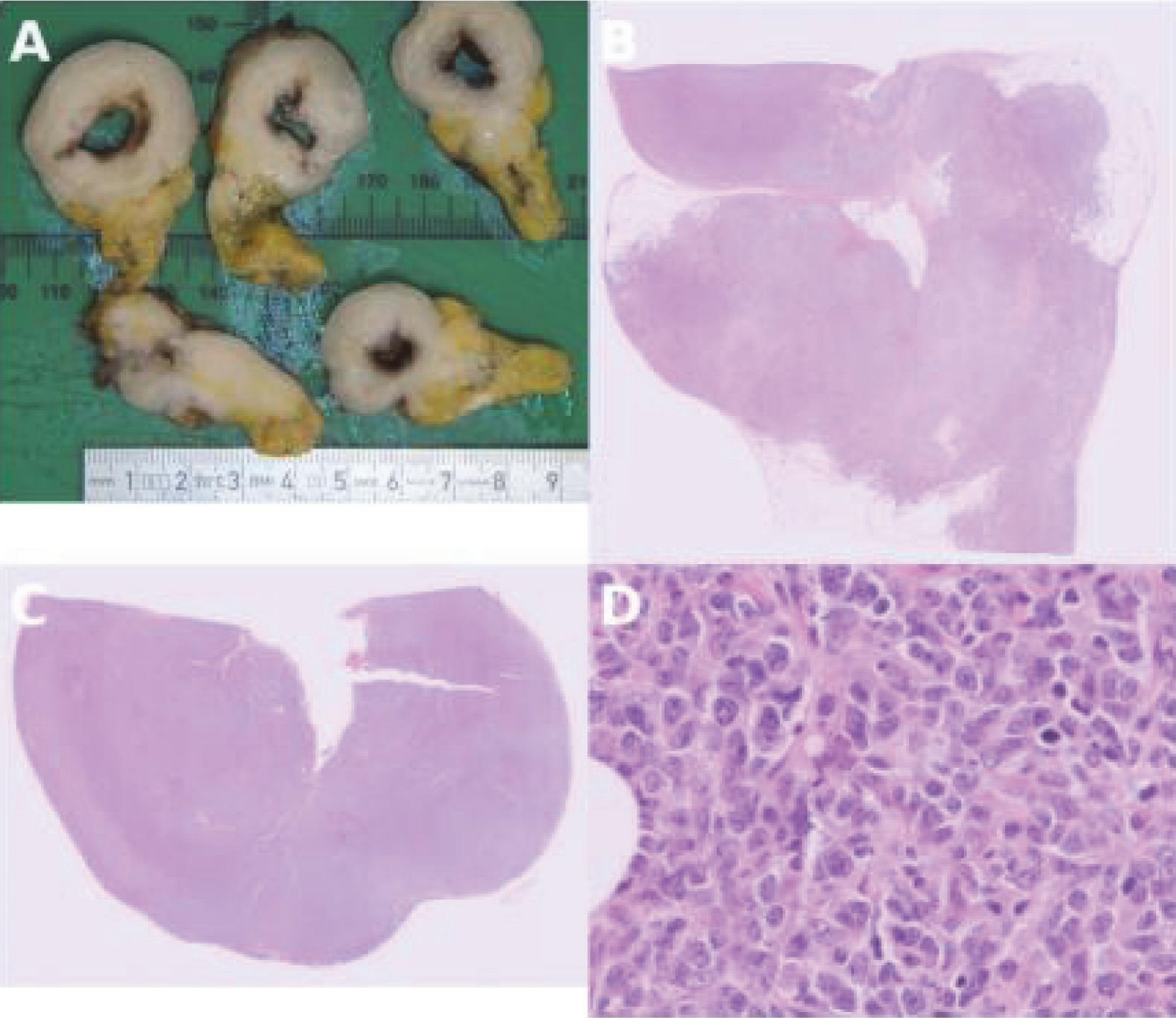
(A) Postoperative small intestinal specimen measuring 150 mm in length, showing a firm, white, markedly thickened wall with severe luminal stenosis and focal near‐complete obstruction. There is complete loss of normal macroscopic small intestinal architecture. (B, C)Histopathological examination of the postoperative specimen demonstrates complete effacement of the normal small‐intestine architecture, replaced by sheets of atypical lymphoid cells, with infiltration into the adjacent mesenteric tissue. H&E staining; original magnification × 10. (D) Sheets of medium‐sized, atypical, lymphoid cells with irregular, pleomorphic nuclei, prominent nucleoli, and numerous mitotic figures. H&E staining; original magnification ×400. Immunohistochemical analysis demonstrated that the tumor cells were positive for CD20, CD10, and BCL6, and negative for BCL2, CD5, MUM1, c‐MYC, cyclin D1, TdT, and EBER. Ki‐67 showed a proliferation index of ~50%. Fluorescence in situ hybridization (FISH) analysis demonstrated loss of one copy of c‐MYC (8q24) but no evidence of c‐MYC rearrangement or “double‐hit” lymphoma.

The patient was referred to hematology and initiated systemic chemotherapy with R‐CHOP‐21 for six cycles, along with appropriate supportive therapy. All six cycles of R‐CHOP‐21 were completed. The patient reported no fever or night sweats. However, he experienced an unintentional weight loss of 14 kg over 2 months during the recurrent obstructive course, most likely related to reduced oral intake. Time‐line is presented in Table 1.

**Table 1:** Clinical timeline of recurrent small bowel obstruction leading to diagnosis of primary intestinal diffuse large B‐cell lymphoma.

Timepoint	Clinical event	Key findings/outcome
Day 0	Initial emergency department presentation	Acute abdominal pain and vomiting. CT demonstrated small bowel obstruction with dilated loops and a transition point in the right lower abdomen.
Day 0–1	Initial inpatient evaluation	Contrast passage study demonstrated propagation to the colon, although residual contrast persisted in dilated small bowel loops, suggesting partial obstruction.
Day 1	Clinical improvement and discharge	Conservative management resulted in passage of stool and flatus; patient discharged without planned follow‐up.
Day 4 (week 1)	First recurrence	Representation with recurrent obstructive symptoms. Repeat CT confirmed recurrent SBO and demonstrated a newly visualized mesenteric soft tissue lesion suspicious for neuroendocrine tumor.
Day 4–9 (week 1)	Repeat inpatient assessment	Persistent small bowel dilatation on contrast study. CT thorax and abdomen demonstrated no distant metastatic disease. Temporary symptom resolution with conservative treatment.
Day 9	Discharge	Clinical improvement following repeat conservative management.
Day 12 (week 2)	Second recurrence	New episode of clinically and radiologically confirmed SBO with increasing mesenteric soft tissue changes adjacent to right‐sided small bowel loops. Prolonged contrast study failed to demonstrate full resolution.
Day 16 (week 3)	Definitive surgical intervention	Exploratory laparotomy with segmental small bowel resection and primary anastomosis. Distal ileal tumor with mesenteric extension identified.
Postoperative week 1	Histopathological diagnosis	Diffuse large B‐cell lymphoma confirmed.
Postoperative week 2	Oncologic treatment initiated	Hematology referral and initiation of systemic R‐CHOP chemotherapy.
Early postoperative period	Emergency reassessment	Re‐presentation with abdominal pain; CT demonstrated a subcutaneous midline abscess.
Postoperative month 1	Early follow‐up reassessment	Repeat CT demonstrated unchanged residual mesenteric soft tissue abnormality without progression.

## 3. Discussion

### 3.1. Diagnostic Significance of Recurrent Small Bowel Obstruction

This case illustrates the diagnostic challenge of recurrent, transiently resolving SBO in a patient without previous abdominal surgery. Although the patient initially improved with conservative management, serial imaging demonstrated a persistent transition point and an increasingly evident mesenteric abnormality, indicating an underlying pathological lead point. The principal clinical message is that temporary symptomatic or radiological improvement does not exclude a structural or malignant cause of obstruction.

Small bowel lymphoma commonly causes nonspecific symptoms, including abdominal pain, weight loss, nausea, and gastrointestinal bleeding [5]. Mechanical obstruction is a less common initial manifestation because lymphoma may infiltrate the bowel wall without producing the marked desmoplastic response or early luminal narrowing typically associated with adenocarcinoma or neuroendocrine tumors [8]. Aggressive lymphomas, particularly DLBCL, may nevertheless produce circumferential bowel wall thickening or a bulky intestinal or mesenteric mass resulting in obstruction [8].

The present case was unusual because the obstruction temporarily resolved on more than one occasion. This may have reflected intermittent mechanical compromise caused by tumor‐associated bowel wall thickening, changing bowel position, and dynamic mesenteric tethering rather than a completely fixed luminal occlusion. The transient improvement contributed to repeated conservative management despite the persistence of the transition point. The subsequent clearer delineation of the mesenteric lesion on CT demonstrates the importance of interpreting serial examinations collectively rather than assessing each episode in isolation.

The initial radiological suspicion of a neuroendocrine tumor also illustrates the potential diagnostic overlap between small intestinal malignancies. Both neuroendocrine tumors and lymphoma may be associated with an intestinal lesion and mesenteric soft tissue abnormality. In this patient, definitive differentiation required operative exploration and histopathological examination.

### 3.2. Systematic Literature Search and Structured Review

To place the present case in context, we conducted a systematic literature search of MEDLINE and Embase followed by a structured review of published adult cases of intestinal lymphoma presenting with SBO on May 18, 2026, in collaboration with a medical librarian (Figure 3). The search combined terms relating to intestinal obstruction, ileus, SBO, lymphoma, non‐Hodgkin lymphoma, DLBCL, and case reports. Eligible publications described adults with clinically or radiologically confirmed bowel obstruction in whom intestinal lymphoma was established histopathologically. Cases were excluded when lymphoma‐directed medical treatment preceded the obstructive presentation, bowel obstruction was insufficiently documented, or adequate information for the eligibility assessment was unavailable. Cases of intussusception were included when accompanied by clinically relevant bowel obstruction. Where publications described multiple patients, eligibility was assessed at the individual case level [12–15].

**Figure 3:**
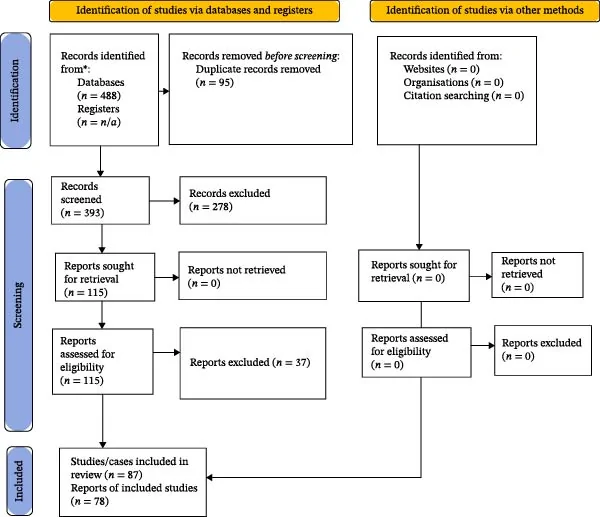
PRISMA flow diagram illustrating the identification, screening, eligibility assessment, and final inclusion of studies evaluating small bowel obstruction as the initial presentation of intestinal lymphoma [11].

The search identified 488 records, comprising 281 from MEDLINE and 207 from Embase. After duplicate removal, 393 records underwent title and abstract screening. Of these, 115 publications were assessed in full text, and 78 publications describing 87 eligible adult patients were included. The literature spanned five decades, from the earliest eligible report published in 1976 [16] to reports published in 2026 [17–20], demonstrating that intestinal lymphoma presenting with SBO has remained an uncommon but repeatedly documented clinical entity. The study‐selection process is presented in the PRISMA flow diagram in Figure S1.

Among the 87 patients, 63 were male (72.4%) and 24 were female (27.6%), corresponding to a male‐to‐female ratio of ~2.6:1. The mean age was 54.2 years, and the median age was 58 years, with a range of 18–89 years. The predominance of male patients should be interpreted cautiously because the literature consisted mainly of individual case reports and small case series and may therefore be affected by publication and selection bias.

The ileum was the most frequently reported anatomical location, accounting for 27.6% of cases (*n* = 24), followed by the jejunum in 23.0% (*n* = 20) and the ileocecal region in 16.1% (*n* = 14). The affected small bowel segment was not specified in 17.2% of cases (*n* = 15). The predominance of distal small bowel involvement may be related to the abundance of gut‐associated lymphoid tissue and Peyer’s patches in the ileum [5]. The ileal location in the present patient was therefore consistent with the most commonly reported anatomical pattern.

DLBCL was the most frequently reported histopathological subtype, accounting for 37.9% of cases (*n* = 33), consistent with previous descriptions of DLBCL as the predominant aggressive B‐cell lymphoma of the gastrointestinal tract (5,15,16). T‐cell lymphoma represented 16.1% (*n* = 14), Burkitt lymphoma 8.0% (*n* = 7), follicular lymphoma 6.9% (*n* = 6), and unspecified non‐Hodgkin lymphoma 6.9% (*n* = 6). Mucosa‐associated lymphoid tissue lymphoma and primary intestinal lymphoma not otherwise specified each accounted for 4.6% (*n* = 4), while plasmablastic lymphoma was identified in 3.4% (*n* = 3) and mantle cell lymphoma in 2.3% (*n* = 2). Rare single‐case subtypes included marginal zone lymphoma, ALK‐positive large B‐cell lymphoma, and post‐transplant lymphoproliferative disorder. These findings support DLBCL as an important lymphoma subtype to consider when an intestinal mass presents with mechanical obstruction.

Intussusception was reported as the obstructive mechanism in 26.4% of cases (*n* = 23), consistent with previous reports identifying lymphoma as a pathological lead point in adult intussusception [13, 16]. Most other cases were attributed to luminal narrowing, circumferential tumor involvement, or mass effect without intussusception. Although several previous reports have described acute SBO as the initial manifestation of intestinal lymphoma [7, 9, 10, 17, 18], recurrent obstruction with repeated temporary resolution, as observed in the present patient, was rarely emphasized. Recent clinical evidence has similarly highlighted intestinal obstruction as an important complication of primary gastrointestinal lymphoma and has demonstrated the relevance of tumor‐related and clinical characteristics in patients developing obstruction [21]. The present case adds to this literature by demonstrating a less frequently emphasized clinical course characterized by recurrent obstruction with temporary symptomatic and radiological improvement.

Surgical intervention was reported in 74.7% of patients (*n* = 65). Segmental bowel resection was the most commonly reported procedure and was performed in 41.4% of cases (*n* = 36). Small bowel resection with primary anastomosis was explicitly reported in 24.1% (*n* = 21), whereas right hemicolectomy or ileocecal resection was performed in 11.5% (*n* = 10). Exploratory laparotomy with biopsy alone was reported in 8.0% (*n* = 7). Less frequently reported procedures included stoma formation and laparoscopic intervention, each in 4.6% (*n* = 4), and intestinal bypass in 1.1% (*n* = 1).

Systemic oncological treatment following diagnosis was explicitly documented in 31.0% of cases (*n* = 27). This figure probably reflects incomplete follow‐up reporting in the included publications rather than the true proportion of patients receiving lymphoma‐directed treatment.

HIV infection or acquired immunodeficiency syndrome was explicitly reported in 3.9% of the included cases, whereas HIV status was not specified in most reports. Inflammatory bowel disease and transplant‐related immunosuppression were also infrequently described. Because relevant comorbidities were inconsistently reported, no firm conclusions can be drawn regarding their prevalence. Nevertheless, the findings indicate that intestinal lymphoma should also be considered in patients without known immunosuppression, as demonstrated by the present case.

### 3.3. Clinical Implications and Management

Recurrent SBO in a patient without previous abdominal surgery should prompt reconsideration of the underlying cause. Although adhesions may occur in a so‐called virgin abdomen, alternative diagnoses—including internal hernia, inflammatory stricture, primary small bowel tumor, metastatic disease, and intestinal lymphoma—should be actively considered [2]. Intermittent symptom resolution should not be interpreted as evidence that the underlying pathological process has resolved.

The present findings do not imply that all patients with transiently resolving SBO require immediate operative intervention. Rather, management should be guided by the clinical course, serial imaging findings, and the likelihood of a persistent pathological lead point. Recurrent symptoms, a persistent transition point, progressive bowel wall thickening, or an evolving intestinal or mesenteric mass should prompt repeat cross‐sectional imaging and multidisciplinary reassessment. Operative exploration should be considered on an individualized basis when malignancy or another structural lesion remains suspected.

Surgery in primary intestinal lymphoma is generally performed to manage complications such as obstruction, perforation, bleeding, or intussusception, or to obtain tissue when a less invasive diagnostic approach is not feasible [8]. Systemic chemotherapy remains the cornerstone of treatment for DLBCL [5]. In the present patient, segmental small bowel resection relieved the obstruction and established the diagnosis, but radical resection was not considered safe because the central mesenteric component encased major vessels. The presence of macroscopic residual disease illustrates why extensive surgery should not be pursued at the cost of major vascular or intestinal morbidity when effective systemic treatment is available.

The role of endoscopy depends on the suspected anatomical location, the patient’s clinical condition, and whether the lesion is accessible. Conventional upper gastrointestinal endoscopy and colonoscopy may help identify additional gastrointestinal involvement but provide limited assessment of most of the jejunum and ileum. In an acutely obstructed patient with a radiologically identified distal small bowel and mesenteric lesion, operative management and histopathological diagnosis may take priority. The rationale for the investigations selected in the present case should nevertheless be stated explicitly in the case presentation.

## 4. Strengths and Limitations

The principal strength of this report is the detailed longitudinal description of recurrent obstructive episodes, including serial CT examinations, operative findings, and histopathological correlation. The structured literature search adds context by demonstrating how frequently different lymphoma subtypes, anatomical locations, and obstructive mechanisms have been described.

The literature review also has important limitations. It was based predominantly on case reports and small case series and is therefore vulnerable to publication bias, incomplete reporting, and clinical heterogeneity. Study selection and data extraction were performed by a single reviewer, which may have increased the risk of selection error and subjective assessment. In addition, no formal risk‐of‐bias assessment was undertaken because the evidence consisted predominantly of individual case reports and small case series. The reported frequencies should be interpreted as descriptive characteristics of published cases rather than population‐based estimates. Treatment and outcome data were inconsistently reported, and the finding that systemic treatment was documented in only 31.0% of cases should not be interpreted as evidence that most patients did not receive chemotherapy. In addition, the search was restricted by language, and two potentially relevant publications could not be assessed because full‐text articles were unavailable.

The present report is further limited by its single‐case design, and the exact mechanism responsible for the intermittent obstruction and apparent radiological variation in tumor size cannot be established definitively. Causal conclusions about the optimal timing of surgery cannot be drawn from this case or from the descriptive literature review.

This case report was prepared and reported in accordance with the SCARE 2025 guidelines [22].

Recurrent SBO in a patient without previous abdominal surgery should raise suspicion of an underlying pathological lead point, even when symptoms temporarily resolve. Serial imaging findings should be evaluated together with the clinical course, and an evolving intestinal or mesenteric abnormality should prompt consideration of malignancy, including intestinal lymphoma. Repeat cross‐sectional imaging, multidisciplinary reassessment, and individualized consideration of operative exploration may facilitate timely diagnosis.

## Author Contributions

Karl Nilsson Grip and Jenny Nyqvist‐Streng conceived the study. Ann‐Catrin Ekelund performed the literature search strategy. All authors contributed to data interpretation, manuscript drafting, revision, and approved the final manuscript.

## Funding

No external funding was received for this work.

## Disclosure

All authors contributed to data interpretation, manuscript drafting, revision, and approved the final manuscript. No ChatGPT, OpenAI, and cloud‐based platform was used for clinical interpretation, literature selection, data extraction, analysis, diagnostic reasoning, or generation of scientific conclusions. All AI‐assisted (ChatGPT, OpenAI, and cloud‐based platform) output was critically reviewed, edited, and verified by the authors, who take full responsibility for the accuracy and integrity of the manuscript.

## Ethics Statement

According to guidance issued by the Swedish Ethical Review Authority, a case report limited to the description of diagnosis, treatment, and clinical course does not constitute research requiring ethical review under Swedish law (Guide to the Ethical Review of Research on Humans, p. 55). Therefore, review and approval by an ethics committee were not required for this case report, and no ethics approval number is applicable. The case report was prepared in accordance with the ethical principles of the Declaration of Helsinki. The patient provided both oral and written informed consent for the publication of the clinical information and accompanying images. Patient confidentiality has been protected throughout the manuscript.

## Consent

Written informed consent for publication was obtained from the patient and is available for review by the Editor‐in‐Chief upon request.

## Conflicts of Interest

The authors declare no conflicts of interest.

## Supporting Information

Additional supporting information can be found online in the Supporting Information section.

## Supporting information


**Supporting Information** Figure S1: Detailed search strategies used for the structured literature review. The supporting file contains the complete electronic search strategies for MEDLINE and Embase, including database‐specific subject headings, free‐text terms, Boolean operators, language restrictions, and the number of records identified at each stage of the search process.

## Data Availability

The data supporting the findings of this study consist of anonymized clinical information from a single patient together with data extracted from previously published studies included in the literature review. Individual patient data are not publicly available due to patient confidentiality but may be made available from the corresponding author upon reasonable request and subject to applicable ethical and legal restrictions. All literature‐derived data are available in the cited publications.
