# Supplementary material for: Recurrent Intermittent Small Bowel Obstruction as an Initial Manifestation of Primary Intestinal Diffuse Large B‐Cell Lymphoma: A Case Report and Systematic Literature Review
Source: Case Rep Surg. 2026 Oct 14;2026:1085579. doi: 10.1155/cris/1085579 (PMC13662958; doi:10.1155/cris/1085579)
Supplement: Supplementary file 1 [file CRIS-2026-1085579-s001.docx]

Ovid MEDLINE(R) ALL

| 1 | exp Intestinal Obstruction/ | 52568 |
| --- | --- | --- |
| 2 | exp Ileus/ | 6528 |
| 3 | ((intestinal adj2 obstruction) or ileus or small bowel obstruction or small-bowel obstruction or SBO).ab,kf,ti. | 40591 |
| 4 | 1 or 2 or 3 | 72152 |
| 5 | exp Lymphoma, Non-Hodgkin/ | 121169 |
| 6 | ((intestinal adj3 lymphoma*) or (gastrointestinal adj3 lymphoma*) or diffuse large B-cell lymphoma or DLBCL or Nonhodgkin* or Non Hodgkin* or Non-Hodgkin's).ab,kf,ti. | 65804 |
| 7 | 5 or 6 | 145571 |
| 8 | 4 and 7 | 612 |
| 9 | exp Case Reports/ | 2552839 |
| 10 | case.ab,kf,pt,ti. | 2735169 |
| 11 | 9 or 10 | 4080511 |
| 12 | 8 and 11 | 421 |
| 13 | 12 not (animal or animals or cadaver or cadavers or cadaveric or cat or cats or cow or cows or dog or dogs or goat or goats or hamster or hamsters or horse or horses or koalas or monkey* or mouse or mice or murine or pig or pigs or porcine or rabbit or rabbits or rat or rats or rodent or rodents or swine).ti. | 412 |
| 14 | 13 not animals/ not (animals/ and humans/) | 409 |
| 15 | 13 not (child or children or pediatric* paediatric* or infant* or neonat*).ti. | 388 |
| 16 | limit 15 to (danish or english or norwegian or swedish) | 281 |

Embase <1974 to 2026 May 14>

| 1 | intestine obstruction/ | 41550 |
| --- | --- | --- |
| 2 | Ileus/ | 19325 |
| 3 | ((intestinal adj2 obstruction) or ileus or small bowel obstruction or small-bowel obstruction or SBO).ab,kf,ti. | 52775 |
| 4 | 1 or 2 or 3 | 86177 |
| 5 | non-Hodgkin lymphoma/ | 78182 |
| 6 | diffuse large B cell lymphoma/ | 41283 |
| 7 | ((intestinal adj3 lymphoma*) or (gastrointestinal adj3 lymphoma*) or diffuse large B-cell lymphoma or DLBCL or Nonhodgkin* or Non Hodgkin* or Non-Hodgkin's).ab,kf,ti. | 118476 |
| 8 | 5 or 6 or 7 | 153866 |
| 9 | 4 and 8 | 783 |
| 10 | case report/ | 3266870 |
| 11 | case study/ | 119543 |
| 12 | case.ab,kf,ti. | 3609926 |
| 13 | 10 or 11 or 12 | 5244632 |
| 14 | 9 and 13 | 456 |
| 15 | 14 not (animal not (animal and human)).sh. | 453 |
| 16 | 15 not (child or children or pediatric* paediatric* or infant* or neonat*).ti. | 443 |
| 17 | limit 16 to (danish or english or norwegian or swedish) | 395 |
| 18 | limit 17 to (article or article in press or conference paper or note or "review") | 207 |

| Medline | 281 |
| --- | --- |
| Embase | 207 |
| Totalt | 488 |
| Dubbletter | 95 |
| Abstrakt kvar att screena | **393** |
